# The Effect of the Severity of Obstructive Sleep Apnea on Leukocyte Telomere Length, 25 Hydroxy Vitamin D, and Parathyroid Hormonal Concentrations in Asian Indians

**DOI:** 10.3389/fneur.2021.682739

**Published:** 2021-10-26

**Authors:** Surya Prakash Bhatt, Randeep Guleria, Naval K. Vikram

**Affiliations:** ^1^Department of Pulmonary, Critical Care and Sleep Medicine, All India Institute of Medical Sciences, New Delhi, India; ^2^Department of Medicine, Metabolic Research Group, All India Institute of Medical Sciences, New Delhi, India

**Keywords:** obstructive sleep apnea, aging, metabolic disorder, Asian Indian, obese, sleep apnea

## Abstract

**Background:** Obstructive sleep apnea (OSA) is a common disorder in which breathing repeatedly stops during sleep. Leukocyte telomere length (LTL) and OSA are linked with an increased risk of oxidative stress and inflammation. The possible link between LTL and OSA in Asian Indians has not been evaluated. Thus, the present study aims to compare the link between LTL and OSA in Asian Indians.

**Methods:** In this study, 300 subjects (120 obese with OSA, 110 obese without OSA, and 70 non-obese without OSA) were included after overnight polysomnography and a fasting blood sample. Clinical, anthropometry, metabolic markers, insulin, 25-hydroxyvitamin D [25(OH) D], and parathyroid hormones (PTH) levels were investigated. LTL was investigated by a QPCR. Univariate and stepwise multivariate linear regression analyses adjusting for age, gender, BMI, and % body fat were conducted while treating LTL as a dependent variable in relation to AHI and other covariates.

**Results:** Obese subjects with OSA had significantly decreased 25(OH)D and increased PTH levels. The mean telomere length (T/S) ratio was significantly shorter in patients with OSA. The adjusted correlation analysis showed that shortening of telomere length correlated with increasing age, apnea-hypopnea index (AHI), oxygen desaturation index, and RDI. Univariate analysis showed that LTL revealed a trend toward a negative correlation with a mean age (β + SE, −0.015 + 0.0006; *p* = 0.01) and positive correlation with AHI [β +slandered error (SE), 0.042 + 0.017; *p* = 0.008]. In the multiple regression analysis, LTL was positively associated with AHI (β + SE, 0.281 + 0.04; *p* = 0.001) after adjusting for age, sex, BMI, and % body fat. Even when adjusted for confounding factors, 25(OH)D, and PTH levels, LTL still was related to AHI (β + SE, 0.446 + 0.02; *p* = 0.05).

**Conclusion:** Our study indicates the presence of an association between LTL and OSA and a significant impact of OSA severity and telomeres shortening in Asian Indians.

## Introduction

Obstructive sleep apnea (OSA) is a common sleep breathing disorder described by repetitive upper airway collapse throughout sleep (1). It is caused by either complete obstruction of the airway or incomplete obstruction, both of which can cause arousal from sleep. The hallmark of OSA is the sleep-related blockage of the upper airway.

The prevalence of OSA is estimated at 4% for men and 2% for women among adults in western countries, but increases to as much as 30–50% among obese subjects (2). A population-based study from North India estimated the prevalence of obstructive sleep apnea syndrome (OSAS) at 3.6% and OSA to be 13.7% (3). Another hospital-based study has estimated the prevalence of OSA and OSAS to be 4.4 and 2.4% in men and 2.5 and 1% in women (3). The prevalence of OSA in the Indian population is three-fold higher in men as compared to women (4). A semi-urban population study among the North Indian population found that 6.2% of the total population were at high risk of OSAS, whereas 33.5% of the obese population was at high-risk of OSAS (5). There is little data regarding the prevalence of OSA in the Indian population.

Previous reports have indicated that the presence and severity of OSA was independently associated with increased risk of oral glucose tolerance, obesity, and insulin resistance (6, 7). Another report has indicated that being overweight, obesity, and OSA are separately associated with metabolic dysfunctions and similarly with systemic inflammation (8).

Telomerase is a crucial enzyme that maintains leukocyte telomerase length (LTL) and cellular duplicate potential. In human subjects, the DNA sequence of telomerase is a tandem repeat of six nucleotides –TTAGGG—that extends ~10–15 kilobases (9). LTL naturally shortens with each cell cycle, and cells with critically shortened telomerase undertake replicative senescence and apoptosis. Further, LTL shortening is accelerated by oxidative stress, inflammation, and cell proliferation (10). Furthermore, shorter telomerase in leukocytes has been established to be associated with the increased occurrence of chronic diseases such as cardiovascular disease (11).

Telomerase activity and LTL have been associated with a multiplicity of health conditions. However, a small number of studies have reported the association between OSA and LTL in the adult population (12–14) and found that patients with OSA had shorter LTL as compared to those without OSA. Furthermore, Kwon et al. (13) and Tempaku et al. (14) have correlated the severity of OSA with telomeres shortening, which further established that increased oxidative stress and inflammation may play an important part in this process. However, no published studies analyzing a possible link between obesity, LTL, and OSA in Asian Indians have been conducted. Therefore, in order to better highlight this knowledge gap, we have investigated the relationship of LTL to the severity of OSA in an Asian Indian population.

## Methods

### Subject Recruitment

In this study, a total of 300 individuals, 120 obese (BMI ≥25 kg/m^2^) with OSA, 110 obese without OSA, and 70 non-obese (BMI 18–22.9 kg/m^2^) were recruited between 2015 and 2020 (13). This study was ethically approved by the Institutional Ethics Committee of the All India Institute of Medical Sciences, New Delhi, India, and written informed consent was taken from each subject. Diagnosis of OSA was by full night polysomnography. Subjects with known or diagnosed diabetes mellitus, who were using drugs or meal replacements for weight loss, taking Calcium, and/or vitamin D supplement currently or within the past 6 months, or had a past and current history or family history of renal stones, were excluded from the study. In addition, individuals who joined in any other investigational drug study in the previous 3 months, had any systemic diseases or were taking any kind of treatment for more than 1 month out of the last 6 months, were diagnosed to have malabsorption or a history suggestive of malabsorption, a history of bariatric surgery, using ultraviolet radiation as part of medical treatment, diagnosed with albinism or having other conditions linked with decreased skin pigmentation, and pregnant and lactating individuals were also excluded from the study.

### Demographic Characteristics, Clinical, and Anthropometric Measurements

Demographic characteristics, blood pressure (systolic and diastolic), and BMI were measured (15).

### Biochemical Analysis

Biochemical measurements included fasting blood glucose (FBG), liver and kidney function tests and lipid profile, serum 25-hydroxyvitamin D [25(OH)D], and parathyroid hormones (PTH) and calcium levels using standard methods as described previously (16). Fasting serum insulin levels and HOMA-IR were also measured (17, 18). The intra-assay coefficient of difference of 25(OH)D, PTH, and calcium was 1.7, 2.1, and 3.1% respectively. Overall, for all the variables the intra- and inter-assay coefficient of variation was <5%.

### Polysomnography

All individuals underwent overnight polysomnography (*Medi palm; Braebon Medical Corp., Canada*) and were categorized according to the apnea–hypopnea index (AHI). The recordings were examined with a 60 s epoch, and sleep periods were recorded according to the standard criteria (19). Diagnosis of OSA was made based on the international classification of sleep conditions (ASDA diagnostic classification steering committee). Breathing occasions were defined permitting to the commonly used clinical standards published by the American Academy of Sleep Medicine Task Force (20). Individuals with AHI <5/h were normal and individuals with AHI ≥5/h were diagnosed to have OSA. Patients having OSA were further categorized as mild OSA (AHI ≥5–15/h), moderate OSA (AHI ≥15–30/h), and severe OHA (AHI ≥30/h).

### Measurement of Leukocyte Telomere Length

A total of 10 mL of whole blood from all individuals was collected in EDTA anticoagulant tubes for the extraction of DNA. The DNA was extracted using a QIAamp extraction kit (*Qiagen, Hilden*, and *Germany*) and stored at −20°C for the additional experiments (21). After DNA isolation, the DNA was quantified and diluted to 50 ng/μL. LTL was analyzed with a quantitative real time PCR that compares telomere repeat sequence copy number (T) to a reference single copy-gene copy number (S) (22, 23). Measurement of determining the relative ratio (T/S ratio) of ng of telomeres (T) to ng of single-copy gene (S) in trial samples using a standard curve. The T/S ratio is comparative to the average telomere length. All examinations were done blinded to case-control status of the subjects.

#### Statistical Analysis

Study data were entered in an Excel spreadsheet (Microsoft Corp, Washington, USA). The distribution of body composition and biochemical, clinical, and anthropometric parameters was confirmed for approximate normality. We used mean ± standard deviation and number (%) to summarize the parameters. The results obtained from the cluster analysis were analyzed using one-way analysis of variance (ANOVA), followed by the Bonferroni *post-hoc* test correlation. Analyses were conducted using the Pearson's linear correlation test to examine potential associations among LTL and other parameters. Univariate and multivariate linear regression analyses were conducted while treating LTL as a dependent variable in relation to AHI and other covariates. In addition, logistic regression was carried out to identify the independent risk factor and to estimate the odds ratio (OR) after adjusting for age, gender, BMI, and % body fat; a 95% confidence interval using Stata 14.0 statistical software (College Station, Texas, USA) was used for data analysis. *P*-value <0.05 has been measured as statistically significant.

## Results

### Demographic, Clinical, Body Composition, and Biochemical Profile

The clinical characteristics, body composition, and biochemical parameters of the study subjects have been summarized in Table 1. The percentage of women and men were similar in all subject groups (*p* > 0.05). Mean age was marginally higher in subjects with OSA, but the differences were not statistically significant. Mean values of BMI (*p* = 0.003), AHI (*p* = 0.001), ODI (*p* = 0.001), fat mass (*p* = 0.0001), and % body fat (*p* = 0.002) were significantly higher in obese subjects with OSA. Similarly, FBG (*p* = 0.004), serum TG (*p* = 0.01), TC (*p* = 0.02), AST (*p* = 0.01), ALT (*p* = 0.03), ALP (*p* = 0.05), fasting serum Insulin (*p* = 0.001), and HOMA-IR (*p* = 0.001) were significantly higher in obese subjects with OSA. Obese subjects with OSA had significantly decreased serum 25(OH)D (*p* = 0.005) and increased serum PTH (*p* = 0.006) levels. In addition, vitamin D deficiency was significantly higher in obese with OSA group.

**Table 1:** Demographic, anthropometry and biochemical profiles investigations.

**Variables**		**Obese with** **OSA (1, *n* = 120)**	**Obese without** **OSA (2, *n* = 110)**	**Non-obese without** **OSA (3, *n* = 70)**	**Overall** ***P*-value**	***P*** **-value**		
						**1 vs. 2**	**1 vs. 3**	**2 vs. 3**
Age (years)		43.5 ± 10.6	42.6 ± 9.6	42.6 ± 8.6	0.32	0.09	0.12	0.54
Sex (*n*, %)	Men	70 (58)	65 (59)	42 (60)	0.82	0.07	0.21	**0.05**
	Women	40 (42)	45 (41)	32 (40)	0.52	0.09	0.65	**0.04**
Body mass index (Kg/m^2^)		29.5 ± 7.9	26.8 ± 6.9	22.4 ± 8.6	**0.003**	0.08	0.06	**0.05**
Fat mass (kg)		41.45 ± 18.5	31.2 ± 12.6	22.3.2 ± 6.3	**0.0001**	**0.0005**	**0.007**	0.5
Body fat (%)		43.2 ± 15.6	33.5 ± 10.6	30.02 ± 10.3	**0.002**	**0.004**	**0.005**	**0.61**
AHI (events/hour)		25.30 ± 6.55	4.45 ± 1.34	2.80 ± 1.11	**0.001**	**0.0001**	**0.0001**	**0.05**
ODI (events/hour)		38.96 ± 7.8	10.97 ± 6.9	7.8 ± 4.3	**0.001**	**0.004**	**0.001**	**0.04**
Total sleep time (minutes)		393.26 ± 54.28	408.42 ± 44.73	406.42 ± 35.7	0.148	0.21	0.004	0.87
Mean sat O_2_ (%)		86 ± 4.5	90 ± 3.45	95 ± 6.98	**0.04**	**0.05**	**0.05**	0.61
Fasting blood glucose (mg/dl)		122 ± 35.6	106.4 ± 30.1	90 ± 17.8	**0.004**	**0.03**	**0.06**	**0.006**
Serum triglycerides (mg/dl)		192 ± 40.6	177 ± 46.9	151 ± 58.9	**0.01**	**0.05**	**0.004**	**0.004**
Total cholesterol (mg/dl)		193.6 ± 38.3	187 ± 44.6	179 ± 39.8	**0.02**	**0.05**	**0.07**	**0.04**
High density lipoprotein (mg/dl)		41.36 ± 8.3	53.6 ± 10.2	55.6 ± 9.5	**0.002**	**0.009**	**0.05**	**0.25**
Alanine transaminase (IU/L)		44.5 ± 15.9	41.4 ± 22.1	31.6 ± 15.9	**0.01**	0.5	**0.04**	**0.002**
Aspartate aminotransferase		60.9 ± 10.3	54.2 ± 12.9	50.9 ± 10.9	**0.03**	**0.04**	**0.03**	0.54
Alkaline phosphate (IU/L)		240.6 ± 74.3	242 ± 76.5	235 ± 69.8	**0.05**	**0.54**	0.62	**0.05**
Fasting insulin (μU/ml)		14 ± 7.2	12.4 ± 6.5	9.5 ± 5.9	**0.001**	**0.04**	**0.05**	**0.04**
HOMA-IR		3.0 ± 0.93	2.3 ± 0.80	1.7 ± 0.80	**0.001**	**0.05**	**0.009**	**0.03**
25-hydroxy vitamin *D* (ng/ml)		13.9 ± 3.8	17.1 ± 7.6	29.8 ± 9.4	**0.005**	**0.05**	**0.006**	**0.008**
Parathyroid hormone (pg/ml)		61.9 ± 12.5	59.6 ± 20.6	41.5 ± 18.3	**0.006**	**0.04**	**0.004**	**0.006**
Calcium (mg/dL)		9.6 ± 0.13	9.4 ± 0.3	9.0 ± 2.5	0.8	0.92	0.56	0.54

*Values are in ± SD. P <0.05 is statistically significant. Boldface values are the statistically significant values. One-way ANOVA and Bonferroni post-hoc test has been done AHI, apnea hypopnea Index; ODI, oxygen desaturation index*.

**Figure 1:**
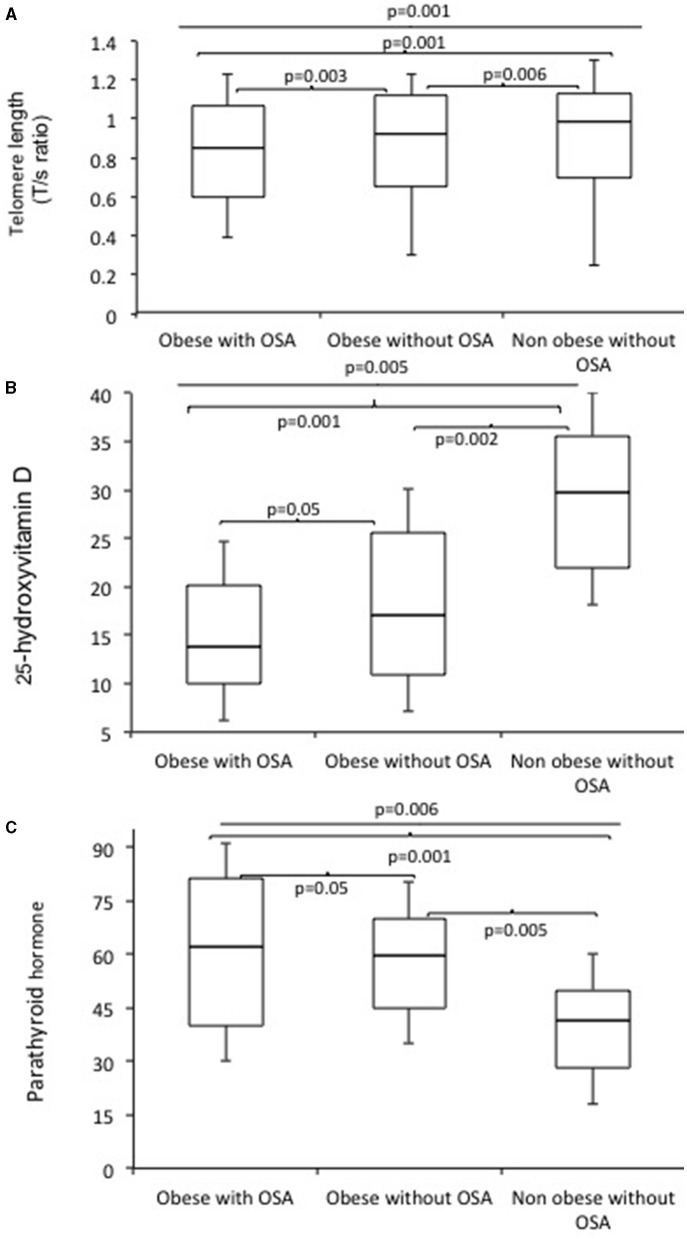
Boxplots of telomerase length (T/S ratio) **(A)**, 25 hydroxy vitamin D **(B)**, and serum parathyroid hormones levels **(C)** in obese subjects with OSA, obese subjects without OSA, and non-obese subjects without OSA.

**Figure 2:**
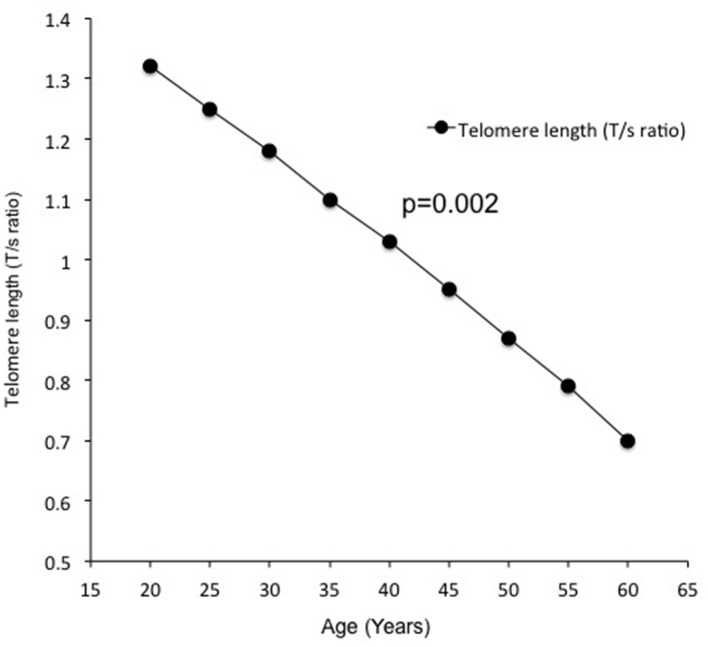
Association of telomerase length with age.

Telomere length measured by the average T/S ratio was significantly shorter in obese subjects with OSA (0.853 ± 0.070) as compared to obese subjects without OSA (0.920 ± 0.82) and non-obese subjects without OSA (0.991 ± 0.090) (Figure 1). This difference persisted after adjustment for age, BMI, FBG, serum TG, CHO, fasting serum insulin, HOMA-IR, 25(OH)D, and PTH (*p* = 0.018). In addition, LTL significantly decreased with increasing age (Figure 2).

### Bivariate and Multivariate Analyses of Factors Related to Telomere Length

The unadjusted correlation analysis displayed that telomere shortening was significantly correlated with age, AHI, ODI, RDI, desaturation Index, minimum SpO_2_, BMI, % body fat, fasting insulin, HOMAIR, 25(OH)D, and PTH (Table 2); we did not get any significant correlations with FBG, serum TG, TC, ALT, AST, and ALP. After adjustment for age, only AHI, ODI, and the desaturation Index were significantly connected to telomere shortening (Table 2).

**Table 2:** Univariate and bivariate analyses to identify factors associated with telomere length.

**Parameters**	**Simple correlation**		**Age and sex adjusted regression**	
	**Correlation coefficient (*r*)**	***P*-value**	**Regression coefficient (95% CI)**	***P*-value**
Age (years)	−0.067	**0.046**	–	–
Sex	−0.08	**0.003**	–	–
Apnea-hypopnea index	−0.199	**<0.001**	0.001 (−0.07 to 0.001)	**0.031**
ODI, events/hours	00.243	**0.003**	0.059 (0.010 to 0.031)	**0.004**
Respiratory disturbances index	−0.150	**<0.001**	−0.03 (−0.07 to 0.01)	0.212
Desaturation index	−0.189	**<0.001**	−0.04 (−0.08 to 0.001)	**0.01**
Minimum SpO_2_	0.211	**<0.001**	−0.02 (−0.06 to 0.02)	0.312
BMI (Kg/m^2^)	−0.143	**0.021**	−0.04 (−0.08 to 0.00)	0.081
Body fat (%)	−0.015	**0.011**	**–**0.03 (−0.07 to 0.01)	0.140
Fasting blood sugar (mg/dL)	0.06	0.231	−0.01 (−0.05 to 0.04)	0.712
Serum triglyceride (mg/dL)	0.010	0.806	0.01 (−0.03 to 0.05)	0.512
Total cholesterol (mg/dL)	0.041	0.461	0.00 (−0.04 to 0.05)	0.912
Alanine transaminase (IU/L)	0.049	0.431	−0.02 (−0.01 to 0.05)	0.913
Aspartate aminotransferase	0.070	0.631	0.01 (−0.02 to 0.08)	0.612
Alkaline phosphate (IU/L)	0.210	0.432	0.004 (−0.07 to 0.06)	0.412
Fasting insulin (μU/ml)	−0.196	**<0.001**	−0.01 (−0.05 to 0.03)	0.511
HOMA-IR	−0.176	**0.002**	−0.02 (−0.07 to 0.02)	0.431
25-hydroxy vitamin *D* (ng/ml)	0.031	**0.04**	0.199 (0.09 to 0.211)	**0.005**
Parathyroid hormone (pg/ml)	−0.186	**0.003**	−0.03 (−0.07 to 0.01)	0.231

*r, Spearman's coefficient; p, statistical significance. Boldface values are the statistically significant values*.

### Regression Analyses

In Univariate analysis, LTL was negatively correlated with mean age [β + standard error (SE), −0.015 + 0.0006; *p* = 0.01] and positive correlation with mean AHI (β + SE, 0.042 + 0.017; *p* = 0.008; Table 3). In the multiple regression analysis, LTL was positively associated with AHI (β + SE, 0.281 + 0.04; *p* = 0.001) after adjusting for age, gender, BMI, and % body fat. Even when adjusted for confounding factors, 25(OH)D, PTH levels, and LTL still was linked with AHI (β + SE, 0.446 + 0.02; *p* = 0.05).

**Table 3:** Univariate and multivariate analyses between AHI and LTL and covariates.

**Independent variable**	**Telomerase length (T/S Ratio)**					
	**Univariate**			**Stepwise multivariate**		
	**β**	**SE**	***p***	**Beta**	**SE**	***p***
Age	−0.015	0.0006	0.011	–	**–**	**–**
Sex	0.006	0.019	0.68	–	**–**	**–**
BMI	0.013	0.009	0.125	–	**–**	**–**
Body fat (%)	0.0110	0.090	0.118	–	**–**	**–**
AHI	0.042	0.017	0.008	0.281	**0.04**	**0.001**

*BMI, body mass index; AHI, apnea hypopnea index; SE, standard error. P value is < 0.05 is statistically significant. Boldface values are the statistically significant values*.

## Discussion

The novel finding of this research is that low LTL and serum 25(OH)D, and high PTH levels are independently related to the incidence of OSA. Additionally, we found a positive association between the AHI and LTL for the entire group. In particular, such self-regulating association of OSA and LTL is being defined for the first time in the Asian Indian population.

Telomerase shortening and dysfunction in cellular senescence has been recognized to promote aging and age-related illnesses. Studies in animal models indicate the potential role of telomerase as an anti-aging effector. In humans, it may have a similar role, but the data are controversial (24). In addition, some studies evaluated that stains and a high Mediterranean diet are associated with higher telomerase activity and lower telomerase shortening in humans (24). The level of leukocyte telomerase activity is important in determining LTL in aging cells and tissues (25). Telomere shortening resulting from the absence of telomerase activity may be an important factor in determining age-related properties of organs in humans. It remains unclear if age-corrected LTL plays an active pathogenic role in the predisposition to adverse outcomes. Short LTL in peripheral-blood mononuclear cells (PBMCs) is associated with aging and aging related illnesses, such as obesity, T2DM, and cardiovascular disease. Some lifestyle interventions, including the Ornish and the Mediterranean diets, have also demonstrated increased leukocyte telomerase activity (26). In this regard, OSA has also been associated with systemic inflammatory and oxidative stress. These associations may increase with the severity of OSA.

Our findings are in concordance with previously published studies describing the association between OSA and LTL in adults (10, 11, 27–29). However, to date, this is the first research specifically considered to investigate the connection between LTL and OSA risk shown in the Asian Indian population. A recent systematic review and meta-analysis included 2,639 participants and concluded that subjects with OSA have smaller LTL, which requires early involvement and timely treatment for avoiding future adverse outcomes (10). Further, a case-control study reported that intermittent hypoxemia was the chief contributor to telomere shortening. In addition, LTL was also correlated with arterial stiffness and increased the risks associated with aging (11). Barceló et al. (28) showed that LTL in circulating leukocytes was shortening more in patients with OSA than without OSA. An epidemiological study (*n* = 1,042) in the Brazilian population observed an independent association of short LTL with long sleep duration (>8 h) and insomnia (29). An immunological study, Aric et al. (30) examined worldwide sleep quality (examined by the Pittsburgh Sleep Quality Index), and diary-reported sleep period with LTL in different immune cell divisions [granulocytes, peripheral blood mononuclear cells, CD8+ and CD4+ T lymphocytes, and B lymphocytes] in 87 obese subjects. They showed that worldwide sleep quality was significantly linked with shorter LTL in CD8+ and CD4+ T lymphocytes (30). Our results also show major significance in aiding the understanding of previous studies by verifying a relationship between telomere shortening and obesity (BMI and fat mass) (31). This association has been speculated to be reflected in adipocytes, which may be apparent as a systemic endocrine pro-aging effect of adipose tissue, particularly visceral adipose tissue (32). However, OSA is a common disorder in obese and overweight subjects and may therefore play an important role in the association relating obesity to LTL.

In this study, patients with OSA showed higher BMI, WC, and other fat-related parameters, and LTL correlated with these parameters by univariate analysis. Importantly, these parameters did not correlate with LTL after adjustment for age and sex, or by multi-variate analysis, which identified ODI as the only significant factor. In addition, we did not find a collective effect of OSA-related intermittent hypoxia and obesity on telomere shortening. Thus, our findings suggest that OSA-related intermittent hypoxia may play an important role to accelerate aging independently of obesity.

Clinical studies have shown the associations between LTL and vitamin D. Richards et al. (33) were the first to establish a negative correlation among serum 25(OH)D and LTL. Williams et al. (34) showed that vitamin D is unlikely to be a significant determinant of LTL, at least by early adulthood. A prospective study in the United States reported that higher 25(OH)D levels may be related with longer LTL length, and this association may be modified by calcium intake (35). It is important to note that only two previous studies, in a limited number of subjects, have explored the effects of vitamin D supplementation on LTL, including a retrospective trial (in a Spanish population). Zhu et al. (36) showed that the rise in serum 25(OH)D levels with vitamin D supplementation was accompanied by a 19.2% increase of leukocyte telomerase activity as compared to a placebo group. Further, Borras et al. (37), showed longer LTL in hemodialysis patients treated with Calcirol for at least 6 months compared to those without treatment. Moreover, serum vitamin D levels were negatively related to DNA methylation aging (38).

Similarly, the results of previous research on Vitamin D, LTL, and OSA have been inconsistent. Based on pre-clinical studies, it has been suggested that OSA is a possible modulator of 25(OH)D and LTL (39, 40), mainly via the effect of intermittent hypoxia on inflammation and oxidative stress.

One of the important limitations of the study includes the small sample size. Further, we did not estimate biomarkers of oxidative stress and inflammation, so we could not examine the possible relationship between them and TL. Furthermore, we did not define the rate of alteration of TL against time, or the possible effects on the action of OSA with continuous positive airway pressure therapy, which might deliver additional insights into the significance of telomere dynamics in OSA patients. Finally, longitudinal and intervention studies are necessary to study the interactions between LTL, vitamin D deficiency, and OSA, and also to assess the influence of improving sleep continuity and treating OSA on natural aging.

## Conclusion

This study suggests that OSA is independently related to the presence of shorter LTL, low serum 25(OH)D, and high PTH levels in Asian Indians.

## Data Availability Statement

The original contributions presented in the study are included in the article/supplementary material, further inquiries can be directed to the corresponding author/s.

## Ethics Statement

The studies involving human participants were reviewed and approved by All India Institute of Medical Sciences, New Delhi, India. The patients/participants provided their written informed consent to participate in this study.

## Author Contributions

SB and RG conceived and designed all the experiments. SB performed the experiments, analyzed the data, and wrote the paper. SB, RG, and NV contributed reagents, materials, and analysis tools. All authors contributed to the article and approved the submitted version.

## Funding

This research study was fully supported by funding (File Number: SB/YS/LS-326/2013) from the Department of Science and Technology, Ministry of Science and Technology, Government of India. The funding agency had no part in study design, data collection, and analysis, decision to publish, or preparation of the manuscript.

## Conflict of Interest

The authors declare that the research was conducted in the absence of any commercial or financial relationships that could be construed as a potential conflict of interest.

## Publisher's Note

All claims expressed in this article are solely those of the authors and do not necessarily represent those of their affiliated organizations, or those of the publisher, the editors and the reviewers. Any product that may be evaluated in this article, or claim that may be made by its manufacturer, is not guaranteed or endorsed by the publisher.

## Abbreviations

OSA: obstructive sleep apnea
LTL: leukocyte telomere length
BMI: body mass index
AHI: apnea-hypopnea index
CI: confidence interval
OR: odds ratio
FBG: fasting blood glucose
25(OH)D: 25 hydroxy vitamin D
PTH: parathyroid hormone
ITT: intention to treat
PP: per protocol
T2DM: type 2 diabetes mellitus
NGT: normal glucose tolerance
HbA1c: hemoglobin
TC: total cholesterol
TG: serum triglycerides
HDL-C: high-density lipoprotein cholesterol
LDL-c: low-density lipoprotein cholesterol
SGOT: serum glutamic-oxaloacetic transaminase
SGPT: serum glutamate pyruvate transaminase.
